# Approaches for Extracting Nanofibrillated Cellulose from Oat Bran and Its Emulsion Capacity and Stability

**DOI:** 10.3390/polym14020327

**Published:** 2022-01-14

**Authors:** Wiphada Mitbumrung, Numphung Rungraung, Niramol Muangpracha, Ploypailin Akanitkul, Thunnalin Winuprasith

**Affiliations:** Institute of Nutrition, Mahidol University, Nakhon Pathom 73170, Thailand; wiphada.mit@gmail.com (W.M.); numphung.run@mahidol.ac.th (N.R.); niramol.mua@mahidol.ac.th (N.M.); ploypailin.aka@mahidol.ac.th (P.A.)

**Keywords:** nanofibrillated cellulose (NFC), chemical pretreatment, hydrothermal pretreatment, oat bran, storage modulus

## Abstract

The pretreatment process is an essential step for nanofibrillated cellulose production as it enhances size reduction efficiency, reduces production cost, and decreases energy consumption. In this study, nanofibrillated cellulose (NFC) was prepared using various pretreatment processes, either chemical (i.e., acid, basic, and bleach) or hydrothermal (i.e., microwave and autoclave), followed by disintegration using high pressure homogenization from oat bran fibers. The obtained NFC were used as an emulsifier to prepare 10% oil-in-water emulsions. The emulsion containing chemically pretreated NFC exhibited the smallest oil droplet diameter (*d*_32_) at 3.76 μm, while those containing NFC using other pretreatments exhibited *d*_32_ values > 5 μm. The colors of the emulsions were mainly influenced by oil droplet size rather than the color of the fiber itself. Both NFC suspensions and NFC emulsions showed a storage modulus (*G*′) higher than the loss modulus (*G*″) without crossing over, indicating gel-like behavior. For emulsion stability, microwave pretreatment effectively minimized gravitational separation, and the creaming indices of all NFC-emulsions were lower than 6% for the entire storage period. In conclusion, chemical pretreatment was an effective method for nanofiber extraction with good emulsion capacity. However, the microwave with bleaching pretreatment was an alternative method for extracting nanofibers and needs further study to improve the efficiency.

## 1. Introduction

Cellulose is a homopolysaccharide consisting of a β-1,4 linked glucopyranose unit which can have a polymerization degree from 300 to 15,000 [1,2]. Nanocellulose, defined as less than 100 nm in diameter, has attracted growing interest for many applications because of its functional properties such as nano size, high surface area, amphiphilic property, low density, high mechanical strength, eco-friendliness, nontoxicity, and low cost [3,4]. Nanocellulose functions as a composite film [5], performs the encapsulation and delivery of vitamin D_3_ [6], serves as a reinforcement material [1], an emulsifier [7], and can be used in energy applications [8], active packaging [8], cosmetics [9], and cellulose nanopapers [10]. Nanocellulose has been classified as three types, including (1) cellulose nanocrystal (CNC)/nanocrystalline cellulose (NCC) which is a rod-like crystalline region cellulose; (2) cellulose nanofibril (CNF)/nanofibrillated cellulose (NFC) which is a long entangled cellulose with amorphous and crystalline regions; and (3) bacterial cellulose (BC) produced from bacteria [11]. In order to extract cellulose from plant sources regarded as lignocellulosic biomass, it is necessary to have a pretreatment process to remove non-cellulosic materials (i.e., lignin, hemicellulose, pectin, and wax) that are embedded within the cellulose structure [12]. Intensive mechanical disintegration is a final step for producing NFC which consumes a lot of energy and time. Mechanical processes, such as grinding or high-pressure homogenizing, are the most common methods used to disintegrate and reduce cellulose fibril sizes [13]. The pretreatment is a significant process to improve the defibrillation of the cellulose, reduce energy consumption, and avoid clogging in the machine from the entanglement of cellulose. Different pretreatment methods (chemical, physical, physicochemical, and biological) have been used for different purposes depending on the source of the cellulose and the preferred type of nanocellulose [14,15]. Alkaline pretreatment is regarded as a chemical pretreatment and is an effective method used for removing the lignin, hemicellulose, and non-cellulosic substances from lignocellulosic materials [13]. Alkaline coupled with acid pretreatment increased the aqueous swelling of cellulose which assists non-cellulosic removal and depolymerization. However, the use of chemicals for the pretreatment of cellulose, such as the neutralization of the pretreated cellulose, and the effects on the environment from chemical waste, chemical recycling, time consumption, and safety for use as food additive were considered [16,17]. On the other hand, chemical-free pretreatment has seen increasing interest in many studies. Hydrothermal pretreatment is a simple and cost-effective physical pretreatment process. Using hydrothermal pretreatment without chemical addition affected physical changes by the re-localization of lignin on the cellulose surface, solubilization of hemicellulose, and increased accessibility of the cellulose structure [18,19].

In general, nanofibrillated cellulose is obtained by extraction from woody plants [1], [20,21,22] or agricultural waste [23,24,25,26]. The processes of purifying cellulose and defibrillating are continually carried out when the cellulose is wet. In this study, the cellulose source for nanofibrillated cellulose production was purified oat bran fiber which contained 98% dietary fiber. Oat bran is a by-product of oat milling which is counted as 50% whole grain. Oat bran is an excellent source of dietary fiber. Oat bran fiber is suited for food application due to its natural source (safety) and neutral taste. The utilization of oat bran as a source of NFC is also a good selection for low cost and massive NFC production [27,28]. The oat bran fiber was in the form of a dried white power. There is a challenge in defibrillating from dried purified cellulose. Many research studies showed that the drying process of cellulose fibers altered the cellulose structure including the molecular packing. When cellulose is in a wet state, water forms hydrogen bonds with -OH groups in the structure of the mobile chain of cellulose, and the cellulose structure is loosely packed. When water is removed, the mobility of the cellulose chain is reduced by increasing the interchain bonds in the cellulose chain, so the cellulose structure is shrunken and densely packed [29,30,31]. The dry cellulose can be rehydrated by the discharging of the interchain bonds which allows water to interact with the cellulose structure. However, the drying process causes some structural changes, and the original swollen state may not be regained because hydrogen bonds in the wet state are irreversible [32]. Due to pretreatment methods (chemical and hydrothermal methods) having effects on the cellulose structure, they may increase the rehydration ability of dry cellulose and also facilitate mechanical stress for defibrillation into nanofibrillated cellulose.

Pickering emulsion was first described by [33,34] as the utilization of solid particles as stabilizers. The stabilization of Pickering emulsion involved the particles being partly wetted by oil and water and the accumulation of the Pickering emulsifier at the oil/water interface which formed a steric barrier against coalescence. The outstanding properties of Pickering emulsions are long-term stability due to the particles being irreversibly adsorbed at the interface which offers stronger attraction than surfactant adsorption, so many of the particles were easily modified at their surface to provide a beneficial property. Moreover, some natural particles displayed safety for in vivo usage according to their very low toxicity, biocompatibility, and environmental friendliness [35,36]. The properties of Pickering emulsion (i.e., emulsion droplet size, viscosity, and flocculation) are dominated by the properties of Pickering particles and the arrangement of the particles at the interface. Pickering emulsions are simply categorized as oil-in-water (O/W) or water-in-oil (W/O) according to the wettability and the contact angle at the interface of the particles. For example, for the particles formed at a contact angle of less than 90°, these particles are likely to be wetted by water which preferred to form an O/W emulsion [37]. Since the trend of using natural emulsifier for stabilizing emulsion has been increasing, cellulose is commonly used due to its advantages over synthetic emulsifiers i.e., biological origin, biocompatibility, biodegradability, renewability, sustainability, and nontoxicity [38,39,40]. Furthermore, the strong points of using cellulose to stabilize emulsions are a high potential to be an emulsifier due to their amphiphilic property which can turn a hydrophobic or hydrophilic side to an optimal surrounding, providing high stability by the packing of dense particle layers at the interface which sterically stabilizes emulsions against droplet coalescence and forming a network owing to the high aspect ratio [41].

In this research, we focused on the effect of the pretreatment process by chemical (alkaline and acid) and hydrothermal techniques including microwave and autoclave treatments to facilitate the mechanical defibrillation of oat fiber and study the emulsion capacity of NFC extracted from dried purified oat fibers. The oat fibers were pretreated with either chemical treatment or hydrothermal treatment followed by bleaching with hydrogen peroxide before being subjected to high-pressure homogenization as mechanical defibrillation to obtain the NFC. The characteristics of NFC were determined including microstructure, color, electrical charge (ζ-potential), and apparent viscosity. The O/W Pickering emulsions were produced using NFC as a single emulsifier by dispersing in the continuous aqueous phase at 1% *w*/*w*. The NFC emulsions were analyzed to assess their properties and stability including emulsion particle size (*d*_32_) with distribution, ζ-potential, color, viscoelastic property, apparent viscosity, and creaming stability.

## 2. Materials and Methods

### 2.1. Materials

Fine type purified oat fiber (98% dietary fiber) extracted from oat spelt bran (JELUCEL-OF90) was obtained from Brenntag Ingredients (Brenntag Ingredients PLC, Bangkok, Thailand). Soybean oil was purchased from local supermarkets and used without further purification. All chemicals were of analytical grade and prepared using deionized (DI) water. Potassium hydroxide (KOH) was obtained from Merck (Merck Co., Ltd., Darmstadt, Germany). 37% Hydrochloric acid (HCl) was obtained from QRëC (Quality reagent chemical Co., Ltd., Esparreguera, Spain). 30% v/v Hydrogen peroxide (H_2_O_2_) was obtained from Chem Supply (Adelaide, South Australia). Dipotassium hydrogen phosphate (K_2_HSO_4_) and potassium dihydrogen phosphate (KH_2_SO_4_) which used to prepare potassium buffer (pH7) and sodium azide (NaN_3_) were obtained from Ajax Finechem (Ajax Finechem Pty., Ltd., New South Wales, Australia).

### 2.2. Oat Nanofibers Extraction

**Figure 1:**
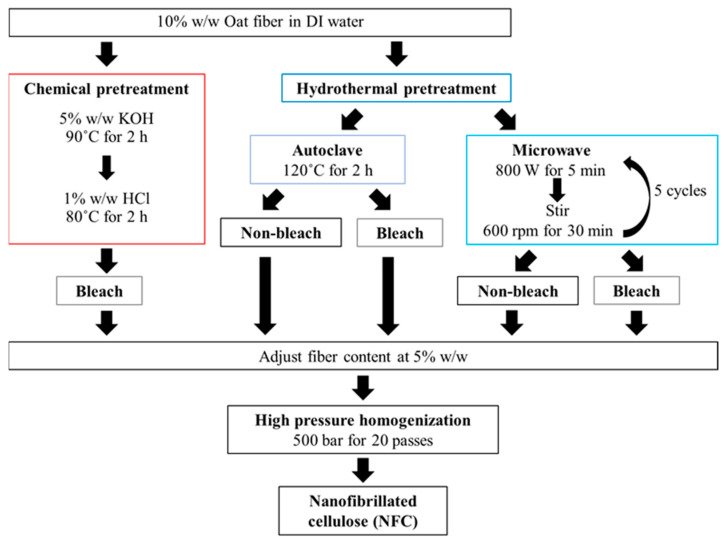
Schematic of nanofibrillated cellulose (NFC) extraction from oat fibers by chemical and hydrothermal pretreatments with mechanical defibrillation by high pressure homogenization.

Nanofibrillated cellulose (NFC) was extracted from oat fibers by chemical and hydrothermal pre-treatments with mechanical defibrillation by high pressure homogenization as shown in Figure 1.

#### 2.2.1. Chemical Pretreatment

Oat fiber was treated with alkaline and acid treatments following the method of [3]. The oat fiber at a concentration of 10% *w*/*w* was dispersed in DI water and then the alkaline treatment was applied to the oat fiber suspension using 5% *w*/*w* of potassium hydroxide (KOH) at 90 °C for 2 h. The oat fiber suspension was then subjected to acid treatment using 1% *w*/*w* hydrochloric acid (HCl) at 80 °C for 2 h. After the chemical pretreatments, the oat fiber suspension was rinsed with DI water and neutralized.

#### 2.2.2. Hydrothermal Pretreatment

The oat fiber suspension at 10% *w*/*w* with DI water was subjected to hydrothermal treatments consisting of either autoclave treatment or microwave treatment following the method of [25,42]. The oat fiber suspension was autoclaved (Autoclave GI54TW, Zealway Instrument INC, Wilmington, NC, USA) at a temperature of 120 °C for 2 h and rinsed with DI water to remove non-cellulosic materials. The oat fiber suspension was also subjected to an 800 W microwave (Sharp R-220, Sharp Thai Co., Ltd., Bangkok, Thailand) for 5 min. After the microwave process, the oat fiber suspension was stirred (Isotemp stirring hotplate, Fisher Scientific International INC, Pittsburgh, PA, USA) at 600 rpm for 30 min and rinsed with DI water to remove non-cellulosic materials. The microwave treatment was repeated for 5 cycles.

#### 2.2.3. Bleaching Process

The oat fiber suspension which was obtained from chemical treatment was bleached with 30% *v*/*v* hydrogen peroxide (H_2_O_2_) at 90 °C for 3 h. The oat fiber suspensions from hydrothermal treatment were either bleached under the same procedure or non-bleached.

#### 2.2.4. Mechanical Process

The solid content of oat bran fiber suspension was adjusted to 5% *w*/*w* using DI water. The oat bran fibers were subsequently defibrillated using the method of [43] who advocated passing the oat fiber suspension through a high-pressure homogenizer (APV-2000, SPX Flow Technology INC, Charlotte, NC, USA) at a pressure of 500 bar for 20 passes. The obtained NFC samples were stored in a water-swollen cellulose state and kept at 4 °C for further study.

### 2.3. Oil-in-Water (O/W) NFC-Stabilized Emulsion Preparation

The O/W emulsions were prepared by mixing 10% *w*/*w* oil phase (soybean oil) and 90% *w*/*w* aqueous phase containing 1% *w*/*w* NFC in 10 mM potassium buffer (pH 7), and 0.01% *w*/*w* sodium azide (NaN_3_). Coarse O/W emulsions were obtained by combining the oil and aqueous phases using a homogenizer (HG-15A equipped with stator dispersing tool HT1025, Daihan Scientific Co., Ltd., Wonju, Korea) at a speed of 10,000 rpm for 2 min. Fine O/W emulsions were obtained by subjecting the coarse O/W emulsions to an ultrasonic sonicator 650 W (Biosafer 650-92 equipped with 6 mm diameter probe, Nanjing Safer Biotech, Co., Ltd., Nanjing, China) at 50% power, pulse on/off 5 s for 5 min. The NFC-stabilized emulsions were kept at room temperature (25 °C) for further analysis.

### 2.4. Scanning Electron Microscope (SEM)

Dried purified oat fiber powder was placed on carbon tape and the microstructure was observed under a scanning electron microscope and energy-dispersive X-ray spectrometer (JSM-IT-300, JEOL, Tokyo, Japan). In the case of water in the NFC suspensions, these were substituted with absolute ethanol prior to being dropped on a glass cover slip. Samples were dried before coating with gold using an ion sputter instrument (SCD 040, Balzers, Bal-Tec GmbH, Pfäffikon, Switzerland). The microstructure of the NFC was then observed under a scanning electron microscope and energy-dispersive X-ray spectrometer (JSM-IT-500HR, JEOL, Tokyo, Japan).

### 2.5. Particle Size and Size Distribution

Particle size (oil droplet diameter) and distribution of freshly prepared NFC-emulsions were measured using a laser particle size distribution analyzer (Mastersizer 3000, Malvern Instruments Ltd., Worcestershire, UK). Emulsion samples were dispersed in DI water to avoid multiple scattering. Refractive indices of oil and water were applied at 1.46 and 1.33, respectively, and absorption was assumed to be 0.

### 2.6. ζ-Potential

NFC suspension at a concentration of 1% *w*/*w* and NFC-emulsions were measured for ζ-potential using a zeta potential analyzer (Zetasizer Nano ZS, Malvern Instruments Ltd., Malvern, Worcestershire, UK). The NFC-emulsions were dispersed in DI water at an oil concentration of 0.6% *w*/*w* prior to measurement.

### 2.7. Color

The 1% *w*/*w* NFC solutions and NFC emulsions were measured for color using a colorimeter with the L*, a*, b* system applied with quartz cuvette (ColorFlex EZ, Hunter Associates Laboratory, Inc., Reston, Virginia, USA). L* represents lightness where L* is 0 = black and L* is 100 = white. Meanwhile, a* and b* represent redness and yellowness where a* + and − values are red and green and b* + and − values are yellow and green.

### 2.8. Rheological Properties

The NFC suspensions (1% *w*/*w*) and NFC-emulsions were measured for viscoelastic properties and viscosity using a controlled-strain rheometer (Physica MCR 302, Anton Paar GmbH, Graz, Austria) equipped with a cone and plate sensor (1° cone angle, 50 mm diameter, and 0.01 mm gap) at room temperature (25 °C). The viscoelastic measurement was conducted by applying a constant strain at 0.5%, with an angular frequency range of 0.1–100 rad s^−1^. The viscosity measurement was taken by increasing the applied shear rate from 0.1–300 s^−1^ for 3 min and decreasing the applied shear rate from 300–0.1 s^−1^ for 3 min.

### 2.9. Creaming Stability

The NFC-emulsions were transferred into a screw-capped glass bottle (20 mm diameter and 70 mm height). The samples were kept under room temperature (25 °C) for 60 days. The creaming stability of the samples was evaluated by observing the phase separation between the serum (bottom) and emulsion (top) phases and calculating the percentage for the creaming index (%CI) via the following equation:

%CI = (H_S_/H_T_) × 100

where H_S_ refers to the height of the serum phase and H_T_ refers to the total height of the emulsion samples.

### 2.10. Statistical Analysis

All measurements were conducted via three replications. The results are shown as mean ± standard deviation. A one-way analysis of variance (ANOVA) with Duncan’s multiple range test at a significance level of *p* ≤ 0.05 was used to indicate significant differences among samples. The statistical analysis was performed using the SPSS version 18.0 Windows program (SPSS Inc., Chicago, IL, USA).

## 3. Results and Discussion

### 3.1. Microstructure of Oat Fibers and NFC

**Figure 2:**
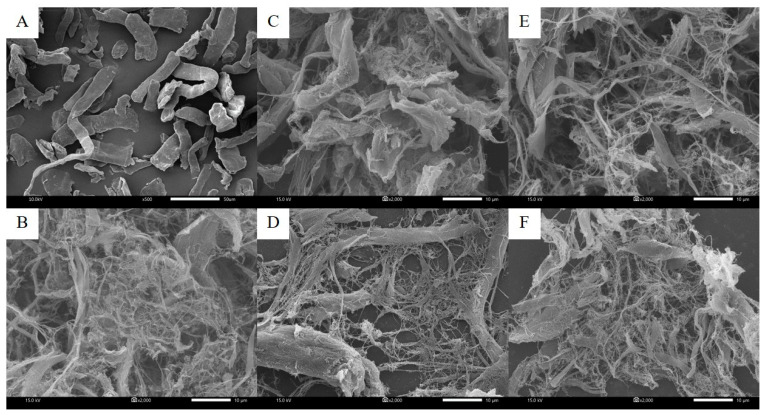
SEM micrographs of the original purified oat fiber (**A**) and NFC pretreated by chemicals (**B**), microwaved without bleaching (**C**), microwaved with bleaching (**D**), autoclave without bleaching (**E**), and autoclave with bleaching (**F**).

The microstructure of the original purified oat fiber and NFC pretreated with chemical and hydrothermal treatments is showed in Figure 2. The shape of the initial oat fiber before extraction is exhibited as densely packed fibers in a cylindrical sheet shape. There was no expansion or entanglement of fine micro/nanofibers. The chemically pretreated NFC exhibited a lot of fine fibers, probably in the nano size range, and the structure of the nanofibers was entangled as a web-like structure. However, there were a few microfibers (≤10 μm diameter) mingled amongst the nanofibers due to inhomogeneity during the defibrillation process. Xiao et al. [3] reported that the cellulose structure obtained by chemical pretreatment separated into individual fibers and formed a three-dimensional network as interconnected fibers. The study of [44] reported in agreement that the alkaline with hydrogen peroxide pretreatment involved the detaching of the cellulose structure which increased the surface area for further processes. The chemical pretreatment greatly facilitated the defibrillation process by high-pressure homogenizer. The microstructures of hydrothermal pretreatments with bleaching and without bleaching were distinguishable. The bleached fibers showed a higher ratio of nanofibers than microfibers and also exhibited web-like entanglement in the structure, while non-bleached fibers exhibited loosely packed microfiber sheets with moderate nanofibers. The result clearly showed that hydrogen peroxide (H_2_O_2_) strongly affected the disclosure of the cellulose structure to enhance the disruption efficiency of the high-pressure homogenizer. The study of [45] found that H_2_O_2_ supplementation for alkaline pretreatment exhibited a higher deterioration of fibrils which indicated that bleaching by using H_2_O_2_ was a significant step for the pretreatment of cellulose. Between two different hydrothermal pretreatments, the fibers obtained from the autoclave were loose and outstretched in contrast to the microwave due to the fact that steam in the autoclave process could better penetrate and severely disintegrate the fiber structure when compared to the microwave process. The microstructure of NFC observed using SEM showed that most fibers were still agglomerated, undivided, and not completely in the form of nanocellulose, which may restrict their dispersion and emulsification properties. These might be caused by the sample preparation step for SEM measurement. The samples had to dry prior to the SEM measurement which caused the structure to collapse and be packed. Somehow SEM may not represent the actual microstructure of the sample in a wet state (either suspension or solution). It is suggested that transmission electron microscopy (TEM) is a more suitable and effective method to observe the microstructure of NFC. In addition, the appropriate concentration of cellulose suspension before subjecting to mechanical defibrillation is also a critical factor affecting the degree of defibrillation. In our study, the oat fiber suspensions were defibrillated at a concentration of 5% *w*/*w* which may restrict the dispersion of cellulose fiber suspension and also obstruct the disintegration step by hindering the disruption force and turbulence flow inside a high-pressure homogenizer. It is suggested that the preparation conditions and process optimizations of several nanocellulose are needed for further study.

The size of nanocellulose is a critical factor indicating emulsifying activity and stability as well as their applications. Unfortunately, the size distribution of NFCs was not included in this study, but the diameter and length of nanocellulose are affected by pretreatment methods and the strength of the mechanical force used for disintegration. The study from [46] showed that NFC from wood pulp by a mechanical method (using a rotor-stator and a microfluidizer) has a diameter of between 20 to 100 nm with a degree of polymerization (DP) at 643, as well as, NFC from wood pulp by chemical pretreatment followed by mechanical method (10%sulfuric acid and microfluidizer) has diameter below 50 nm with DP at 304. It is suggested that size of NFC extracted with chemical pretreatment and mechanical methods provided a smaller size and lower DP which is the same agreement as microstructure showed in this study. Paschoal et al. [47] reported that NFC extracted from oat hull using bleaching, acid hydrolysis (63.7% sulfuric acid), and ultrasonication exhibited interconnected webs of tiny nanofibers with diameters of 70–100 nm and lengths of several micrometers. Furthermore, hydrolysis time did not influence the morphology of extracted NFC. It is also supported that chemical pretreatment before mechanical disintegration could facilitate the breakdown of cellulose fiber.

### 3.2. Particle Size and Distribution

**Figure 3:**
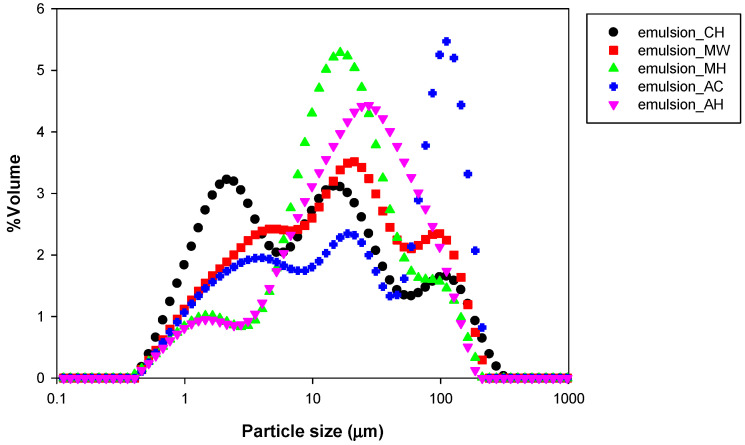
Particle size distribution of emulsions stabilized by NFC pretreated using chemical (CH) and hydrothermal treatments including microwave without bleaching pretreatment (MW), microwave with bleaching pretreatment (MH), autoclave without bleaching pretreatment (AC), and autoclave with bleaching pretreatment (AH).

The particle size of NFC emulsions was reported as a *d*_32_ value (volume–surface mean diameter) which is sensitive to the presence of small oil droplets. As mentioned, this study used NFC as the single emulsifier for O/W stabilization. The NFC itself is a cellulose molecule which stabilized emulsion via the Pickering mechanism. Pickering emulsions (particle-stabilized emulsions) were mainly stabilized by covering the solid particles around the oil droplet surface and forming a steric barrier to prevent droplet coalescence [35,41]. Typically, the oil droplet size presented in Pickering emulsions stabilized by nano-size solid particles was a micrometer in diameter [48]. The results in Table 1 showed that the emulsions stabilized with NFC were in micro size as a common Pickering emulsion. The chemically pretreated NFC emulsion exhibited the smallest oil particles (3.76 ± 0.06 μm). The hydrothermal pretreated NFC emulsion using a microwave with bleaching exhibited larger oil particles compared to non-bleaching. This phenomenon occurred in autoclave pretreatment of NFC emulsion, and it was found that autoclave-pretreated NFC tended to produce bigger emulsion particles than microwave-pretreated NFC. The largest oil particle size was found in emulsion stabilized with bleached autoclaved NFC at 7.28 ± 0.015 μm. The particle size distribution of all emulsions exhibited as multimodal distributions which had 3 peaks in the distribution curve within a range of 1 to 100 μm (Figure 3), because each NFC contained both micro and nano size fibers. Moreover, the difference ratio of microfibers and nanofibers affected the emulsion oil droplet size with the observation that all emulsions produced by NFC contained a heterogeneous oil droplet size. Albert et al. [36] mentioned that the particle size of the Pickering emulsifier influences the emulsion droplet size and stability because the emulsifier size influences the ability to adsorb at the interface during emulsion formation. The nanofibers had the effect of adsorbing at the oil droplet surface rather than microfibers due to their molecular size. Considered from the distribution curve, the chemically pretreated NFC emulsion exhibited smaller oil particle sizes than those hydrothermally treated NFC emulsions because of the higher ratio of nanofibers contained in chemically pretreated NFC. For the same reason, the bleached hydrothermally pretreated NFC emulsions (both microwave and autoclave) exhibited smaller oil particle sizes than non-bleached samples.

### 3.3. ζ-Potential

ζ-potential was used to define the charge of NFC suspensions and the stability of the NFC-stabilized emulsions. ζ-potential of NFC emulsions and NFC suspensions are shown in Table 1 and Table 2, respectively. The ζ-potential of nanocellulose is influenced by extraction methods due to the involvement of ionic chemicals and exposure of COO- in the nanocellulose structure. Originally, cellulose is an anionic polysaccharide that shows a negative charge. In this study, oat bran NFCs with various pretreatments also exhibited negative charge which varied from −17 mV to −27 mV. Overall, the ζ-potential of NFCs in this study exhibited similar values as many previous studies which used different sources of cellulose such as wood and hemp [13], eucalyptus [2], and fluff pulp from commercial paper mills [49]. Moreover, the charge of the NFC dominated the charge of NFC emulsions. In general, the polysaccharide emulsifier stabilized the emulsion by forming a thick interface which stabilized the emulsion through steric repulsion [50]. Since NFC itself exhibited electrical properties, it also stabilized emulsion droplets through electrostatic repulsion. All NFC emulsions exhibited ζ-potential between −37 to −54 mV which pointed to stable emulsions. Interestingly, emulsions stabilized with NFC pretreated by bleached–microwave and bleached–autoclave treatment exhibited very large magnitudes compared with NFC emulsions with unbleached microwave and autoclave pretreatment. The reason was that the hydrogen peroxide used for bleaching revealed the cellulose structure, hence the anionic group (COO-) on cellulose was pronounced.

### 3.4. Color

**Figure 4:**
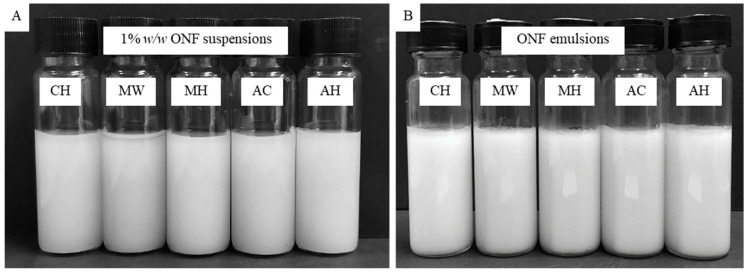
Physical appearance of NFC suspensions (**A**) and freshly prepared emulsions stabilized by NFC (**B**) pretreated using chemical (CH) and hydrothermal treatments including microwave without bleaching pretreatment (MW), microwave with bleaching pretreatment (MH), autoclave without bleaching pretreatment (AC), and autoclave with bleaching pretreatment (AH).

The color parameters of NFC emulsions and NFC suspensions are shown in Table 1 and Table 2, respectively. The appearance of NFC exhibited a white suspension by visual observation. Lightness (L*) of NFC treated with bleaching tended to be higher than unbleached NFC. The color (a* and b*) of all NFC exhibited a sparse yellow even though there were significant differences, but these could not be detected by the human eye (Figure 4). The color of emulsions performed by NFC also exhibited white color as the NFC color. Not only the color of the NFC influenced the color of emulsions, but also the oil particle size contained in the sample had an impact on the emulsion color. Since the color measurement is based on the principle of light scattering, small oil particles have the ability to scatter light rather more than large oil particles. Thus, emulsion containing small oil droplets tended to have a higher L* than emulsion containing large oil droplets. For this reason, the emulsions containing small oil droplets were the chemically treated NFC emulsion and the hydrothermal without bleaching-treated NFC emulsions. Moreover, chemically treated NFC emulsions exhibited little yellowness among all emulsions. In addition to NFC suspensions, NFC emulsions showed no detectable color difference by appearance, although they were significantly different according to the L*, a*, and b* values.

### 3.5. Rheological Properties

**Figure 5:**
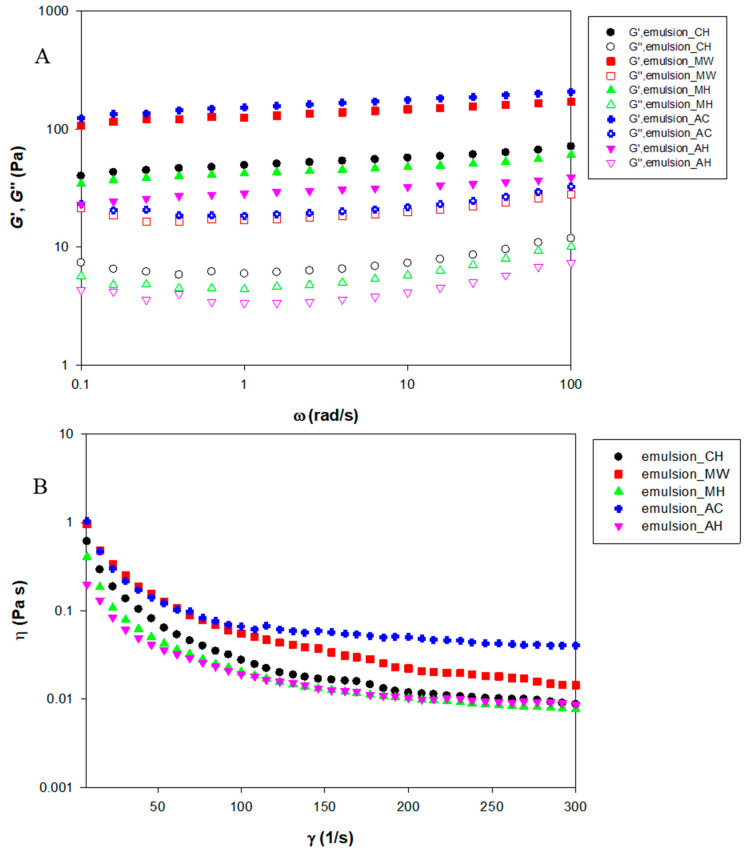
Viscoelastic property (**A**) and viscosity (**B**) of emulsions stabilized by NFC pretreated using chemical (CH) and hydrothermal treatments including microwave without bleaching pretreatment (MW), microwave with bleaching pretreatment (MH), autoclave without bleaching pretreatment (AC), and autoclave with bleaching pretreatment (AH).

The viscoelastic properties and viscosity of NFC-emulsions are shown in Figure 5. In the case of dilute emulsion (low droplet volume fraction), the viscosity of the emulsion was influenced by the viscosity of the continuous phase [51]. The viscosity of NFC emulsions, regarded as dilute emulsions, conformed to the viscosity of the NFC suspension as shown in Table 1 and Table 2. The viscosities of the NFC suspensions pretreated chemically and by hydrothermally without bleaching were not significantly different. The NFC suspensions with bleaching (microwave and autoclave) showed significantly decreased viscosity which may be due to the detachment of the fibrils. However, there were no differences found in the viscosity of NFC emulsions produced by different pretreatment methods. Interestingly, the emulsions stabilized by NFC exhibited lower viscosity than NFC suspensions. The reason was that NFC emulsions had higher magnitudes of ζ-potential. Taheri and Samyn [52] mentioned the decrease of viscosity related to an increase in the ζ-potential value. The repulsion force by ζ-potential acted as a lubricant which caused surface slip [53]. Therefore, the high surface charge emulsions stabilized by NFC treated with microwave with bleaching and autoclave with bleaching exhibited lower viscosity than others. The viscosity of the NFC emulsions pointed out the shear thinning behavior (apparent viscosity decreased as shear rate increased) which is a typical behavior in emulsions occurring via the deformation and disruption of flocs [54]. Since the viscosity of NFC emulsions was influenced by the viscosity of the NFC that presented in the continuous phase, the viscoelastic property was also dominated by the NFC. The NFC emulsions exhibited a *G*’, or storage modulus (referring to a solid-like property), that was over the *G*”, or the loss modulus (referring to a liquid-like property), without crossing over the measured angular frequency range (0.1–100 rad s^−1^) which was a characteristic of the gel-like property. Due to an expansion of the NFC in the continuous phase, not only was viscosity provided to the NFC emulsions but was a three-dimensional network which exhibited gel-like properties was also formed. The study of [55] reported that the emulsion prepared with microfibrillated cellulose (MFC) from mangosteen rind exhibited gel-like behavior. The reasons were: (1) the formation of a three-dimensional gel network from MFC in the continuous aqueous phase of emulsion, and (2) the flocculation of oil droplets induced by fibers. As observed in oil particle size distribution (Figure 3), the emulsion stabilized by autoclaving without bleaching NFC exhibited higher flocs than others. Therefore, it tended to exhibit stronger gel behavior than others.

### 3.6. Creaming Stability

**Figure 6:**
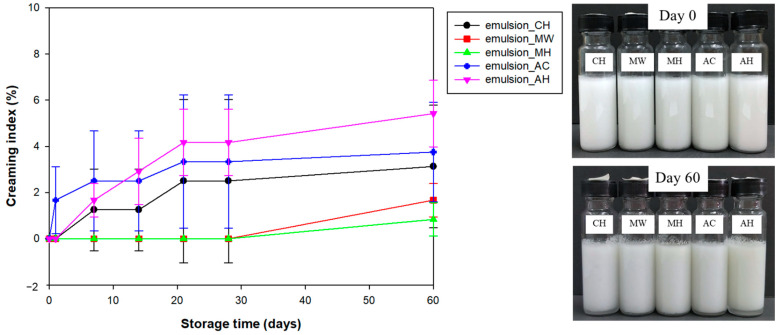
Creaming stability and visual phase separation of emulsions stabilized by NFC pretreated using chemical (CH) and hydrothermal treatments including microwave without bleaching pretreatment (MW), microwave with bleaching pretreatment (MH), autoclave without bleaching pretreatment (AC), and autoclave with bleaching pretreatment (AH).

The creaming index (%*CI*) of NFC emulsions is shown in Figure 6. The creaming index was used to describe instability of the emulsions by observing gravitational separation. The emulsion layer would be located above the serum layer according to their densities. It was possible that the large oil droplet would move upwards leading to the loss of emulsifier wall integrity which caused the oiling off phenomenon. The results showed that the emulsion prepared with autoclave-pretreated NFC would exhibit separation from day 1 at 1.67 ± 1.44%. On day 7, the emulsion prepared with autoclave with bleaching pretreatment and the chemically pretreated NFC started to cream at 1.67 ± 0.72% and 1.25 ± 1.77%, respectively. The emulsions prepared using an autoclave treated with and without bleaching, and chemically pretreated NFC were slightly extended until completing the storage period (60 days) at 3.13 ± 2.65%, 5.42 ± 1.44%, and 3.75 ± 2.17%, respectively. Remarkably, the emulsions using microwave with and without bleaching pretreated NFC started to cream after day 28 and exhibited final %*CI* at 0.83 ± 0.72% and 1.67 ± 0.72%, respectively. The microwave-pretreated NFC decelerated the phase separation rate which improved the long-term stability of the emulsions. All NFC emulsions exhibited relatively low phase separation (less than 6% of *CI*) and no evidence of oiling off was found. This could indicate that emulsions produced by NFC expressed good performance in terms of stability due to the ability of NFC to retard droplet movement by its entangled network located in the continuous phase, the thickening effect, and electrostatic repulsion. It is suggested that key parameters indicating emulsion stability are oil droplet size, interfacial layer at oil–water interfaces (ζ-potential and storage modulus, *G*’), and viscosity of continuous phase. The chemically pretreated NFC emulsion exhibited the smallest oil droplet size, but it showed lower stability than the microwave with bleaching-pretreated NFC emulsion due to lower ζ-potential magnitude, storage modulus, and viscosity. On the other hand, the autoclave with bleaching-pretreated NFC emulsion exhibited an excellent ζ-potential magnitude, but it had the biggest oil droplet size together with the lowest storage modulus, thus, it showed the lowest gravitational stability.

## 4. Conclusions

Nanofibrillated cellulose (NFC) was extracted from purified oat fibers using five different pretreatment methods, including chemical (potassium hydroxide, hydrochloric acid, and hydrogen peroxide), microwave with and without hydrogen peroxide, and autoclave with and without hydrogen peroxide, before being subjecting to a high-pressure homogenizer to defibrillate the fiber. The chemical pretreatment and hydrothermal pretreatment with hydrogen peroxide significantly affected the properties of NFC and its emulsion capacity. Our findings point out that the bleaching step by hydrogen peroxide facilitated the mechanical defibrillation step observed by SEM micrograph. The results found the detaching of fibers and increasing of nano size fibers. However, NFC pretreated with either chemical or hydrothermal methods exhibited micro- and nano-size fibers in various ratios. The NFC-stabilized emulsion was prepared through the Pickering mechanism (solid-stabilized emulsion) by means of steric (forming a thick interfacial layer) and electrostatic (providing a negative charge) repulsions. The chemically pretreated NFC exhibited desirable emulsion capacity by producing the smallest emulsion droplet size. All NFC emulsions exhibited multimodal distribution due to the heterogeneous fiber size of NFC having different capacities to adsorb at the interfacial layer. The ζ-potential indicated that the emulsions stabilized by NFC were stable by sufficient electrostatic repulsion. The colors of the NFC emulsions were influenced by the NFC color and emulsion droplet size. The rheological properties of NFC emulsions were strongly influenced by NFC. Since NFC formed three-dimensional networks in the continuous phase, it increased viscosity and also exhibited the gel-like properties of NFC emulsions. The creaming stability showed that NFC pretreated with microwave with bleaching showed the lowest phase separation which indicated the long-term stability. Even though the NFC with chemical pretreatment facilitated the defibrillation process and exhibited good emulsion capacity and moderate emulsion stability, the NFC pretreated with microwave with bleaching showed similar emulsion capacity and stability. Therefore, microwave with bleaching would be an alternative pretreatment process which is environmentally friendly, reduces the processing time, and involves a mild reaction.

## Figures and Tables

**Table 1:** The mean particle size (*d*_32_), ζ-potential, colors (L*, a*, b*), and apparent viscosity (at shear rate 300 1 s^−1^) of emulsions stabilized with NFC pretreated by chemical and hydrothermal treatments.

Emulsions	*d*_32_ (μm)	ζ-Potential (mV)	L*	a*	b*	η_γ__=300_ (Pa·s)
emulsion_CH	3.76 ± 0.006 e	−39.82 ± 1.51 b	51.56 ± 0.22 ab	−0.11 ± 0.01 a	−0.04 ± 0.03 e	0.0088 ± 0.0003 a
emulsion_MW	5.28 ± 0.015 d	−37.00 ± 2.06 b	52.04 ± 0.01 a	−0.09 ± 0.00 b	0.43 ± 0.02 c	0.0144 ± 0.0006 a
emulsion_MH	6.70 ± 0.010 b	−54.90 ± 3.34 a	49.23 ± 0.67 c	−0.05 ± 0.01 c	0.85 ± 0.08 a	0.0077 ± 0.0001 a
emulsion_AC	6.00 ± 0.012 c	−37.05 ± 1.77 b	51.35 ± 0.11 b	−0.12 ± 0.01 a	0.30 ± 0.05 d	0.0407 ± 0.0109 a
emulsion_AH	7.28 ± 0.015 a	−54.85 ± 3.40 a	49.25 ± 0.22 c	−0.09 ± 0.01 b	0.65 ± 0.04 b	0.0087 ± 0.0023 a

Results were presented as mean ± SD. In the same column, the values with the different letters (a–e) were significantly different from the others (*p* ≤ 0.05). Note: CH—chemical pretreatment; MW—microwave without bleaching pretreatment; MH—microwave with bleaching pretreatment; AC—autoclave without bleaching pretreatment; AH—autoclave with bleaching pretreatment.

**Table 2:** The ζ-potential, colors (L*, a*, b*), and apparent viscosity (at shear rate 300 1 s^−1^) of NFC suspension pretreated by chemical and hydrothermal treatments.

Nanofibrillated Cellulose (NFC)	ζ-Potential (mV)	L*	a*	b*	η_γ__=300_ (Pa·s)
NFC_CH	−20.12 ± 5.28 cd	36.76 ± 0.13 c	−0.76 ± 0.01 a	−4.11 ± 0.02 a	0.2413 ± 0.1227 a
NFC_MW	−26.73 ± 1.41 ab	38.56 ± 0.51 b	−0.71 ± 0.01 b	−3.38 ± 0.06 c	0.2413 ± 0.1963 a
NFC_MH	−23.08 ± 3.72 bc	40.09 ± 0.20 a	−0.70 ± 0.01 b	−3.53 ± 0.02 b	0.0250 ± 0.0045 b
NFC_AC	−27.95 ± 3.58 a	39.29 ± 0.56 ab	−0.78 ± 0.02 a	−3.56 ± 0.02 b	0.2453 ± 0.0412 a
NFC_AH	−17.05 ± 1.94 d	39.80 ± 0.56 a	−0.72 ± 0.01 b	−3.52 ± 0.04 b	0.0288 ± 0.0134 b

Results were presented as mean ±SD. In the same column, the values with the different letters (a–d) were significantly different from the others (*p* ≤ 0.05). Note: CH—chemical pretreatment; MW—microwave without bleaching pretreatment; MH—microwave with bleaching pretreatment; AC—autoclave without bleaching pretreatment; AH—autoclave with bleaching pretreatment.

## Data Availability

Not applicable.

## Abbreviations

CH	Chemical pretreatment
MW	Microwave without bleaching pretreatment
MH	Microwave with bleaching pretreatment
AC	Autoclave without bleaching pretreatment
AH	Autoclave with bleaching pretreatment
